# The Spillover of Socio-Moral Climate in Organizations Onto Employees’ Socially Responsible Purchase Intention: The Mediating Role of Perceived Social Impact

**DOI:** 10.3389/fpsyg.2021.668399

**Published:** 2021-07-08

**Authors:** Marlies Schümann, Maie Stein, Grit Tanner, Carolin Baur, Eva Bamberg

**Affiliations:** ^1^Department of Work and Organizational Psychology, Beuth University of Applied Sciences Berlin, Berlin, Germany; ^2^Department of Work and Organizational Psychology, Universität Hamburg, Hamburg, Germany; ^3^Leadership Excellence Institute Zeppelin, Zeppelin University, Friedrichshafen, Germany

**Keywords:** socio-moral climate, perceived social impact, work-consumption spillover, work-nonwork, employee participation, socially responsible purchase intention

## Abstract

Due to the pressing environmental and social issues facing the global economic system, the role of organizations in promoting socially responsible behavior among employees warrants attention in research and practice. It has been suggested that the concept of socio-moral climate (SMC) might be particularly useful for understanding how participative organizational structures and processes shape employees’ prosocial behaviors. While SMC has been shown to be positively related to employees’ prosocial behaviors within the work context, little is known about the potential spillover effects of SMC (i.e., associations between SMC and employees’ prosocial behaviors outside the work context). The present study aims to address this gap by investigating how and why SMC is related to employees’ socially responsible purchase intention. Drawing on the relational job design framework, we argue that employees’ perceptions of their social impact may explain why SMC is positively related to responsible purchase intentions. We collected data from 492 employees working in various industries at two measurement points with a time lag of 12 months. Hypotheses were tested using path analysis, in which we controlled for the temporal stability of the study variables. The results showed that SMC was positively related to perceived social impact and socially responsible purchase intention and that perceived social impact was positively related to socially responsible purchase intention. In addition, we found a significant indirect relationship between SMC and socially responsible purchase intention through perceived social impact. The findings provide initial support for the spillover of employees’ work-related experiences onto their responsible purchase intentions within the nonwork domain. This study contributes to the literature by extending the traditional focus of SMC research on the development of moral reasoning skills to suggest that perceived social impact is an important mechanism underlying the relationship between SMC and prosocial behaviors. In terms of practical implications, this study suggests that organizational interventions designed to increase SMC may enhance employees’ perceptions of their social impact.

## Introduction

In light of the environmental and social issues in the global economic system, an important question concerning both researchers and practitioners is how organizations can promote socially responsible behavior among their employees. An increasing body of research has addressed this issue by examining the effects of ethical organizational climate (Newman et al., 2017) and socially responsible organizational initiatives (Aust et al., 2020; van Dick et al., 2020) on prosocial behavior and decision-making within organizations. Recently, it has been suggested that the concept of socio-moral climate (SMC; Weber et al., 2009) might be particularly useful for understanding how organizational structures and processes shape employee prosocial behavior (e.g., Pircher Verdorfer and Weber, 2016; Steinheider et al., 2020). Integrating principles of communication, cooperation, and conflict resolution, SMC is closely linked to employees’ participation in discursive-democratic decision-making in organizations and their prosocial behaviors, such as organizational citizenship behavior, workplace solidarity, and democratic engagement (Weber et al., 2009; Pircher Verdorfer et al., 2013).

While several studies indicate that SMC is related to prosocial behavior *within* organizations, little is known about how SMC affects employees’ prosocial behavior *outside* the work context. This is a major gap, considering that understanding the spillover of SMC on employees’ prosocial behavior beyond organizational boundaries may broaden our view of how organizations can meet their social responsibilities. Responsible purchasing, which refers to consumers’ purchase intentions to minimize harmful effects and maximize positive impacts on society in the long term (Mohr et al., 2001), is an important form of prosociality in the nonwork domain. In fact, all employees are consumers, making more or less responsible purchase decisions in their private lives. A spillover of SMC onto employees’ socially responsible purchase intentions would highlight the societal and macroeconomic significance of a participative organizational climate that stimulates employees’ prosocial behavior.

**Figure 1:**
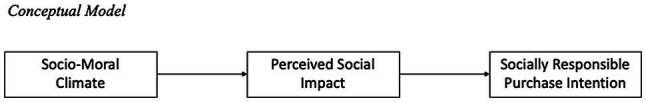
Conceptual model.

This study aims to move beyond understanding the effects of SMC within the organizational context and investigate the relationship between SMC and employees’ socially responsible purchase intention. We combine Grant’s (2007) relational job design framework and learning-generalization theory (Kohn and Schooler, 1973) to gain an in-depth understanding of the process by which SMC is related to employees’ socially responsible purchasing. The relational job design framework links job design and prosocial employee behavior, focusing on the psychological experience of perceived social impact (Grant, 2007). As a reflection of the extent to which employees are aware that their work impacts other people’s lives (Grant, 2007; Bolino and Grant, 2016), perceived social impact is an important driver of prosocial employee behavior (Grant, 2008a,b). By linking employees’ experiences in the work context to their broader attitudes and beliefs underlying their behavior in other contexts, the concept of perceived social impact is particularly useful for investigating the potential spillover of SMC on employees’ socially responsible purchase intention. We suggest that work environments characterized by SMC contribute to the perception of social impact among employees, which in turn affects the extent to which they intend to make responsible purchase decisions. Figure 1 shows the conceptual model.

This study contributes to the literature in three significant ways. First, by integrating research on SMC with the responsible purchasing literature, we advance the understanding of how organizational climate is related to employees’ behavioral intentions in other life domains. Extant research examining the effects of SMC on prosocial employee behavior has focused on the organizational context (e.g., Weber et al., 2009; Schnell et al., 2013; Pircher Verdorfer et al., 2015). By examining prosocial behavioral intentions outside the organizational context, we contribute to the understanding of the spillover effects of SMC. Second, by investigating the mediating role of perceived social impact, we add a different perspective to the theoretical conceptualization of SMC and advance the understanding of why SMC is related to employees’ prosocial behavior. Traditionally, the rationale for expecting SMC to promote employees’ prosocial behavior is that the work environment facilitates the development of moral reasoning skills (Weber et al., 2009). However, empirical research did not support such an underlying process (Pircher Verdorfer and Weber, 2016). By investigating employees’ perceived social impact, we integrate an alternative mechanism that may explain the link between SMC and prosocial behavior. Finally, previous findings on the relationship between SMC and prosocial behavior derive almost exclusively from cross-sectional studies (Weber et al., 2009; Pircher Verdorfer et al., 2013, 2015), making it difficult to draw firm conclusions about the effects of SMC on employees’ prosocial behavior. Conducting a two-wave study, we contribute to the understanding of how SMC is related to employee prosocial behavior over time.

## Theoretical Background

### Socio-Moral Climate and Employee Participation

Socio-moral climate (Weber et al., 2009) refers to “employees’ perceptions of organizational practices and procedures, including principles of communication, teamwork, and participative decision-making assumed to shape the development of ethical attitudes and value orientations of the members of an organization” (Pircher Verdorfer et al., 2015, p. 233–234). According to Pircher Verdorfer et al. (2013, 2015), SMC comprises five components: (1) open confrontation with conflicts of interests, values, and norms, (2) reliable appreciation, including mutual care, respect, and support, (3) cooperation and communication as equals, including opportunities to challenge established norms, (4) trust-based assignment of responsibility for the wellbeing of others inside and outside the organization, and (5) organizational concern for individuals, including the extent to which the organization adopts the perspective of individual employees.

The concept of SMC is rooted in research on democratic participation in organizations. Weber et al. (2008) postulated that employees’ substantial involvement in organizational decision-making nurtures perceptions of a SMC in organizations that facilitates prosocial behavioral orientations. From a theoretical perspective, the notion of moral socialization in organizations induced by democratic participation is based on the just community approach of moral education (Power et al., 1989; Power and Higgins-D'Alessandro, 2008). Inspired by educational structures and principles in Israeli kibbutzim, the just community approach is based on practices of deliberative democracy, including consensus seeking through sustained dialog and participation of all (Power and Higgins-D'Alessandro, 2008). Through its structures of deliberation and accountability, the just community approach is thought to support a sense of responsibility, solidarity, and civic engagement among community members (Power and Higgins-D'Alessandro, 2008). This goal is to be achieved by fostering a moral atmosphere in the community (Power et al., 1989). Drawing primarily on Lempert’s (1988, 1993) work, Weber et al. (2008) adapted this concept for the organizational context, emphasizing its structural dimension of organizational democracy. Specifically, it is proposed that SMC evolves within democratic organizational structures (Pircher Verdorfer and Weber, 2016), forming a socializing context for employees’ social and moral actions (Weber et al., 2009).

### Socio-Moral Climate and Perceived Social Impact

Research has commonly investigated SMC as an antecedent of employees’ positive affective-motivational states toward their organization, including their sense of psychological ownership (Steinheider and Pircher Verdorfer, 2017), work engagement (Steinheider et al., 2020), and organizational commitment (Weber et al., 2009; Pircher Verdorfer et al., 2013). In this study, we draw on the relational job design framework (Grant, 2007) to argue that SMC increases employees’ perceptions of social impact. The job design framework is useful in understanding why work contexts affect employees’ prosocial motivation and behavior.

At the heart of the relational job design framework is the idea that perceived social impact is the psychological mechanism linking job design and employee prosocial behavior. Perceived social impact refers to the recognition that one’s efforts at work make a positive difference in the lives of others (Grant, 2007; Bolino and Grant, 2016). This focus on employees’ work-related experiences and the fundamentally social nature of the concept distinguishes perceived social impact from related concepts, such as self-efficacy. Whereas perceived social impact refers to the *experience* that one’s actions at work have succeeded in benefiting others, self-efficacy concerns the *belief* in one’s capability to achieve desired outcomes and does not speak to the idea that one’s work is benefiting others. Conceptually, the relational job design framework suggests that employees who perceive that their actions have a positive impact on others are more likely to seek to benefit others (Grant, 2007). As the definition of SMC includes employees’ perceptions of cooperation, mutual care, and responsibility for the wellbeing of others, we suggest that SMC strengthens the perception of social impact because organizations characterized by SMC offer their employees various opportunities to have a positive impact on others. Specifically, work environments characterized by SMC involve frequent prosocial interactions in the workplace (Weber et al., 2009; Pircher Verdorfer et al., 2013), which may enable employees to see how their actions benefit others.

Socio-moral climate is inherently participative in that it involves employees having opportunities to contribute and influence. Although varying in the degree of actual influence in decision-making (Dachler and Wilpert, 1978), employee participation describes a process that focuses on employees’ needs and gives them a voice (Earley and Lind, 1987). A participative climate allows employees to experience that they have the power to influence their environment (Spreitzer, 1996). A recent meta-analysis indicates that a participative work climate promotes prosocial behavior within organizations (Weber et al., 2020). Moreover, Lee et al. (2019a) investigated the effects of participative human resource practices and found a positive association between information sharing in organizations and perceived social impact. These findings suggest that opportunities for prosocial influence might be more salient for employees experiencing higher levels of SMC, thus promoting their perception of social impact. Drawing on the relational job design framework and the empirical findings mentioned above, we hypothesize the following:

*Hypothesis 1*: SMC is positively related to employees’ perceived social impact.

### Perceived Social Impact and Socially Responsible Purchase Intention

According to the relational job design framework (Grant, 2007), perceived social impact is necessary for prosocial behavior to occur. Prosocial behaviors refer to various actions that are intended to protect or promote the wellbeing and welfare of other individuals or groups (Bolino and Grant, 2016; Ma et al., 2017; Jung et al., 2020), such as neighbors, coworkers, or the superordinate group of humankind (Reese and Kohlmann, 2015; Costa Pinto et al., 2020). When employees feel that their actions at work have a positive impact on others, they are more likely to engage in behavior that positively impacts the lives of others (Grant, 2007). Empirical support for this notion comes from studies with employees in the airline industry (Ho and Wu, 2019) and the military service (Castanheira et al., 2016). Employees who perceive that they can positively influence others through their work feel closer and emotionally attached to others (Grant, 2008a), promoting prosocial behavior toward them (Korchmaros and Kenny, 2001). Experimental research provides evidence for such an effect for different prosocial work behaviors. For example, enhancing perceived social impact was found to increase employees’ efforts to collect money for those in need among employees of a fundraising organization (Grant et al., 2007) and the helpfulness of lifeguards toward guests (Grant, 2008b).

When employees repeatedly perceive that they can positively influence the lives of others at work, they should become increasingly convinced that their actions generally have a positive impact. This notion is in line with learning-generalization theory (Kohn and Schooler, 1973), suggesting that experiences in the organizational sphere translate into nonoccupational realities. Extant research has found evidence for such a learning-generalization effect, indicating that people learn from experiences in their jobs and generalize what they have learned to nonwork realities (Spenner, 1988). Abundant empirical support for this proposition also stems from research on work-family spillover, which has revealed that experiences at work or in the family domain (e.g., support, conflict, and satisfaction) have a considerable impact on behavior, psychological functioning, and wellbeing in the respective other domain (Ford et al., 2007; Beigi et al., 2019). In support of this view, research on bottom-up processes indicates that domain-specific evaluations may generalize over time to global evaluations. For example, it has been shown that task-specific and occupational self-efficacy beliefs predict subsequent changes in general self-efficacy beliefs (Miyoshi, 2012; Grether et al., 2018). Utilizing this bottom-up perspective, we argue that the work-related perception of social impact generalizes over time to a more global perception of social impact in areas of life other than work.

The perception of social impact implies the awareness that one’s actions indeed have consequences for the lives of others (Grant, 2007). Awareness of consequences is central to the definition of moral decision-making (Schwartz, 1968) and has been highlighted as a key component in influential models of ethical behavior (Schwartz, 1977; Rest, 1984). Being aware of the overall consequences of their actions for the lives of others is necessary for people to feel responsible for engaging in prosocial actions. In the specific case of socially responsible purchasing, awareness of one’s own impact on others is particularly important. Individuals who intend to engage in responsible purchasing aim to mitigate the harm and exploitation of humans, animals, and the environment through their purchase decisions (Burke et al., 2014). Given that these goals can only be achieved if many people purchase responsibly, consumers commonly overlook or deny their own contribution and influence (Schlaile et al., 2018). Conversely, a strong sense of consumer effectiveness has been shown to facilitate responsible consumerism (e.g., Cojuharenco et al., 2016). Based on these considerations, we suggest that the perception of social impact is related to prosociality in the form of socially responsible purchase intention. Specifically, we hypothesize the following:

*Hypothesis 2*: Employees’ perceived social impact is positively related to their socially responsible purchase intention.

### Socio-Moral Climate and Socially Responsible Purchase Intention

According to Karasek’s (2004) idea of job socialization, workplaces are social environments in which adult learning processes occur, and the effects of these learning processes are not limited to the work domain but extend to other areas of life. This is consistent with the conceptualization of spillover as an intraindividual contagion process that occurs across different roles (Bolger et al., 1989; Carlson et al., 2019). For example, employees with opportunities for social interaction at work may develop conversational skills, which are also helpful for conciliation between friends or family members. Work environments characterized by SMC likely contribute to the perception of social impact among employees, which may stimulate socially responsible purchasing in their private sphere. Previous studies that investigated spillover effects of SMC provide support for the potential of SMC to initiate socialization and suggest that SMC is related to employee prosocial behavior. In a cross-sectional study, SMC was shown to be related to community-related prosocial attitudes, indicating that the experience of socio-moral practices at work may generalize to other contexts (Weber et al., 2009). Another cross-sectional study found that SMC was positively related to employees’ ethics-related attitudes and behavioral orientations beyond their pre-occupational childhood socialization experiences (Pircher Verdorfer et al., 2013). We extend this line of research by suggesting that SMC affects employees’ prosocial behavioral intentions outside the work context. In particular, we hypothesize that SMC has an indirect effect on employees’ socially responsible purchase intention through changes in their perceptions of social impact.

*Hypothesis 3*: SMC is positively and indirectly related to employees’ socially responsible purchase intention *via* perceived social impact.

## Materials and Methods

### Sample and Procedure

We tested the hypotheses using a two-wave study design with a time lag of 12 months. This time lag is widely considered to be an appropriate period to capture socialization (Bauer et al., 1998; Woodrow and Guest, 2020). Participants were recruited *via* a German online panel platform. The use of an online panel allowed us to collect a large and heterogeneous sample. Online panel data are comparable to data collected *via* conventional methods in terms of reliability and validity (Buhrmester et al., 2011; Walter et al., 2019). The participants had to answer all items to complete the survey. To ensure data quality, we followed recommendations for detecting careless responders (Meade and Craig, 2012) and excluded participants based on their response time (< 2 s per item), response consistency (even-odd consistency), and bogus items. Of the 605 employees who participated at T1, 113 participants were excluded due to careless responding. Of the remaining 492 employees at T1, 191 employees completed the questionnaire at T2, yielding a response rate of 39%.

At T1, 48% of the respondents were female. Participants’ mean age was 41.8 years (*SD* = 10.8). A total of 27% held a bachelor’s degree or better, and 46% had a secondary school diploma or less. A total of 51% of the participants had a monthly net income of up to 1,500 Euros minus rent. The mean weekly working hours were 38.4 (*SD* = 10.0), and 26% of the participants held supervisory positions. Approximately half of the sample (49%) had worked in their organization for at least 5 years, and approximately two-thirds (65%) worked in the private (vs. public) sector. At T2, the mean age was 45.5 years (*SD* = 10.6). A total of 51% of employees had an income of up to 1,500 Euro minus rent. The mean weekly working hours were 37.5 (*SD* = 10.3), and 22% of the participants held supervisory positions.

### Attrition Analysis

To explore whether there was differential attrition, we conducted a series of *t*-tests and chi-square tests using sociodemographic variables and the study variables. We compared the employees who participated at T1 and T2 with those who participated at T1 only and found no differences between the samples in terms of socially responsible purchase intention [*t*(391.11) = −1.59, *p* = 0.11], perceived social impact [*t*(408.45) = −0.71, *p* = 0.47], SMC [*t*(389.68) = 0.04, *p* = 0.97], working hours [*t*(392.07) = −1.49, *p* = 0.14], supervisory position [*χ*^2^(1) = 0.29, *p* = 0.59], sector [*χ*^2^(2) = 0.61, *p* = 0.73], education level [*χ*^2^(7) = 12.05, *p* = 0.10], tenure [*χ*^2^(2) = 2.52, *p* = 0.28], income [*χ*^2^(4) = 2.01, *p* = 0.73], and gender [*χ*^2^(1) = 0.04, *p* = 0.84]. However, those who participated at T1 and T2 were slightly older (*M* = 44.71, *SD* = 10.36) than the dropouts (*M* = 39.94, *SD* = 10.68), *t*(413.51) = 4.91, *p* < 0.01.

### Measures

The German versions of the scales are provided in the Appendix.

#### Socio-Moral Climate

Socio-moral climate was assessed with the validated 21-item version of the SMC scale (Pircher Verdorfer et al., 2015) developed by Weber et al. (2009). The scale measures the five components of SMC: (1) open confrontation with conflicts (e.g., “In our organization, we deal openly with conflicts and disagreements”), (2) appreciation and respect (e.g., “Our employees are treated with respect regardless of their qualifications or position”), (3) open communication and participative cooperation (e.g., “In our organization, everyone has a voice on important organizational matters”), (4) allocation of responsibility (e.g., “In our organization, everyone is challenged according to his/her skillset”), and (5) organizational concern (e.g., “Although difficult, our organization attempts to meet the needs of all its members”). The items were rated on a 5-point scale with response options ranging from 1 (*strongly disagree*) to 5 (*strongly agree*). Cronbach’s alpha was 0.95.

#### Perceived Social Impact

We assessed perceived social impact with three items developed by Grant (2008b). A sample item is “I feel that I can have a positive impact on others through my work.” The response scale ranged from 1 (*strongly disagree*) to 7 (*strongly agree*). Cronbach’s alphas were 0.92 at both T1 and T2.

#### Socially Responsible Purchase Intention

Socially responsible purchase intention was measured using an adapted version of the six-item Consciousness for Fair Consumption Scale developed and validated by Balderjahn et al. (2013). In the original version, the Consciousness for Fair Consumption Scale is computed by multiplying the scores of the two subscales believe (e.g., “I only buy a product if I believe that in its production the workers’ rights were adhered to.”) and importance (e.g., “How important is it for you personally that in companies the workers’ rights were adhered to.”). We used a different introduction to reflect the actual buying situation that consumers find themselves in by incorporating a tradeoff between social and utilitarian product attributes (e.g., “I would spend more money on products if I had information that in product manufacturing the workers’ rights were adhered to.”). This approach reduces the potential bias due to social desirability. The response scale ranged from 1 (*strongly disagree*) to 7 (*strongly agree*). Cronbach’s alphas were 0.96 at T1 and 0.98 at T2.

#### Control Variables

We decided to include gender as a control variable in the analyses because women have been found to be more willing to consume responsibly than men (e.g., Costa Pinto et al., 2014). Additionally, gender is associated with perceptions of and behavior in prosocial contexts (Croson and Gneezy, 2009).

### Data Analysis

Data were analyzed using path modeling as implemented in the lavaan package (Rosseel, 2012) using the statistics software R (R Core Team, 2019). Model fit was evaluated using the Yuan-Bentler scaled chi-square (*χ*^2^) statistics (Yuan and Bentler, 2000), the comparative fit index (CFI), the Tucker-Lewis index (TLI), the root mean square error of approximation (RMSEA), and the standardized root mean residual (SRMR). CFI and TLI values close to 0.90, RMSEA values less than 0.06, and SRMR values less than 0.08 indicate an acceptable fit of the model with the data (Hu and Bentler, 1999). Maximum likelihood estimation with robust standard errors is used.

In testing Hypothesis 1, we estimated the effect of SMC at T1 on perceived social impact at T2 while controlling for perceived social impact at T1. To evaluate Hypothesis 2, we tested the effect of perceived social impact at T1 on socially responsible purchase intention at T2, controlling for the effect of socially responsible purchase intention at T1. We followed Little’s (2013) recommendations for testing indirect effects using two measurement points. Path *a* of the indirect effect represents the effect of SMC at T1 on perceived social impact at T2 (controlling for its temporal stability), and path *b* represents the effect of perceived social impact at T1 on socially responsible purchase intention at T2 (controlling for its temporal stability). To test for mediation, we estimated unstandardized indirect effects (*ab*) and 95% bias-corrected confidence intervals derived from 1,000 bootstrap samples (BCa CI; Davison and Hinkley, 1997).

In longitudinal research, missing data are inevitable (Jeličić et al., 2009). Ignoring missing data may produce biased estimates, potentially leading to erroneous conclusions. Multiple imputation is a state-of-the-art missing data technique that helps avoid bias (D. A. Newman, 2014). While there is some debate about how to handle whole-wave missing data (Young and Johnson, 2015), multiple imputation has been shown to outperform listwise and pairwise deletion, even when the amount of missing data is large (D. A. Newman, 2014; Madley-Dowd et al., 2019) and data are not missing at random (MNAR; Mustillo and Kwon, 2015). We imputed missing data at the item level for all variables included in the model. We performed multivariate imputation by chained equations using the mice package in R (Van Buuren and Groothuis-Oudshoorn, 2011). In addition to the study variables, we included age, supervisory position, and weekly working hours as auxiliary variables in the imputation model. We used Bayesian linear regression to impute missing data in the continuous variables and logistic regression for the binary variables. The number of imputed datasets was set to *m* = 100. The fraction of missing information values ranged between 0.44 and 0.47, indicating an adequate level variability between imputed data sets (Madley-Dowd et al., 2019).

## Results

Table 1 shows the means, standard deviations, and intercorrelations of the study variables. Socio-moral climate was positively related to perceived social impact and socially responsible purchase intention at T1 and T2. In addition, we found positive correlations between perceived social impact and socially responsible purchase intention at T1 and T2. As expected, the correlations among the same variables over time were relatively high, indicating high temporal stabilities. For the control variable gender, there was a significant positive correlation only for socially responsible purchase intention at T1.

**Table 1:** Descriptive statistics, reliabilities, and bivariate correlations of the study variables.

S. No.		*M*	*SD*	1	2	3	4	5	
1	Socio-moral climate T1	3.29	0.83	0.95					
2	Perceived social impact T1	5.05	1.47	0.40^***^	0.92				
3	Perceived social impact T2	5.13	1.41	0.35^***^	0.59^***^	0.92			
4	Socially responsible purchase intention T1	5.15	1.53	0.09^*^	0.18^***^	0.27^**^	0.96		
5	Socially responsible purchase intention T2	4.95	1.54	0.16^*^	0.26^**^	0.34^***^	0.66^***^	0.98	
6	Gender^a^	0.48	0.05	−0.04	0.01	−0.12	0.11^*^	0.07	

Pearson correlation coefficients: T1/T1: *N* = 492; T1/T2 and T2/T2: *N* = 191. *M*, mean; *SD*, standard deviation. Cronbach’s alphas are given along the diagonal.

a 0 = male; 1 = female.

* *p* < 0.05;

** *p* < 0.01;

*** *p* < 0.001, two-tailed.

Following the recommendations of Becker et al. (2016), we conducted the analyses with and without gender as a control variable. The analyses revealed that the path coefficients were essentially the same for the models with and without control variables,^1^ indicating that gender did not account for the findings. Therefore, we report only the findings of the model without gender. The fit indices indicated a good fit of the hypothesized model with the data: *χ*^2^ (1) = 10.08, *p* < 0.01; TLI = 0.86; CFI = 0.99; SRMR = 0.04; and RMSEA = 0.14, 90% CI (0.07, 0.22). While the RMSEA value is above the generally recommended threshold of 0.06 (Hu and Bentler, 1999), other model fit indices indicate an acceptable model fit. We note that using the RMSEA value for evaluating model fit in this case is problematic given that the RMSEA has been shown to falsely indicate poor fit for models with small degrees of freedom (Kenny et al., 2015). Table 2 shows the results of the path model. The significant path *a* (*b* = 0.29, *SE* = 0.07, 95% CI [0.15, 0.42]) indicates that SMC at T1 was positively related to perceived social impact at T2, and the significant path *b* (*b* = 0.09, *SE* = 0.04, 95% CI [0.01, 0.17]) shows that perceived social impact at T1 was positively related to socially responsible purchase intention at T2. Thus, the results supported Hypotheses 1 and 2. The significant direct effect *c’* (*b* = 0.22, *SE* = 0.07, 95% CI [0.08, 0.36]) indicates a positive relationship between SMC (T1) and socially responsible purchase intention (T2). Hypothesis 3 predicted that SMC is indirectly associated with socially responsible purchase intention *via* perceived social impact. The results showed that the indirect effect of SMC on socially responsible purchase intention *via* perceived social impact (*ab*) was significant (*b* = 0.03, *SE* = 0.01, 95% CI [0.01, 0.05]). Thus, Hypothesis 3 was also supported. Finally, the total effect (*c*) was significant and positive (*b* = 0.25, *SE* = 0.07, 95% CI [0.11, 0.39]).

**Table 2:** Results of path analyses of the hypothesized model.

	Perceived social impact (T2)					Socially responsible purchase intention (T2)				
	*Est*	*SE*	*z*	95% CI	*β*	*Est*	*SE*	*z*	95% CI	*β*
SMC (T1)	0.29^***^	0.07	4.26	[0.15, 0.42]	0.17	0.22^**^	0.07	3.09	[0.08, 0.36]	0.12
PSI (T1)	0.51^***^	0.04	13.67	[0.44, 0.59]	0.53	0.09^*^	0.04	2.12	[0.01, 0.17]	0.08
SRPI (T1)						0.63^***^	0.04	17.91	[0.56, 0.70]	0.60

Pooled estimates of 100 imputed datasets. Sample size after imputation: *N* = 492. SMC, socio-moral climate; PSI, perceived social impact; SRPI, socially responsible purchase intention; and *β*, standardized regression coefficient.

* *p* < 0.05;

** *p* < 0.01;

*** *p* < 0.001.

To explore the potential effects of multiple imputation on the study results, we performed a supplementary sensitivity analysis, in which we included only complete cases. The results of the analysis showed that the path coefficients were very similar, except for the direct effect which was somewhat higher when using the multiply imputed data (path *a* (*b* = 0.28, *SE* = 0.10, 95% CI [0.09, 0.48]), path *b* (*b* = 0.11, *SE* = 0.06, 95% CI [0.00, 0.23]), direct effect path *c’* (*b* = 0.12, *SE* = 0.10, 95% CI [−0.08, 0.32]), indirect effect ab (*b* = 0.03, *SE* = 0.02, 95% CI [−0.01, 0.07]), and total effect *c* (*b* = 0.15, *SE* = 0.10, 95% CI [−0.04, 0.34]).

## Discussion

This study aimed to investigate the spillover effects of SMC on employees’ prosocial behavior outside the work domain in the form of socially responsible purchase intention *via* perceived social impact. Linking the relational job design framework (Grant, 2007) and learning-generalization theory (Kohn and Schooler, 1973) with research on SMC, we have sought to contribute to a deeper understanding of the processes by which SMC is related to employees’ responsible purchasing. Analyses of two-wave data revealed that SMC was positively related to perceived social impact and that perceived social impact was positively related to socially responsible purchase intention over a period of 1 year. In addition, the indirect effect of SMC *via* perceived social impact on socially responsible purchase intention was significant.

### Theoretical Implications

This study advances research on SMC by providing evidence for a positive relationship between SMC and perceived social impact over the course of 1 year. Longitudinal investigations of SMC are scarce. To the best of our knowledge, only one study has examined the effects of SMC using more than one measurement point and found that SMC was positively related to employees’ exercise of individual character strengths at work over a period of 6 months (Höge et al., 2020). We found that SMC was positively related to perceived social impact over 1 year. This result advances the understanding of how SMC affects employees’ prosocial behavior over time. In its original conceptualization, SMC is assumed to stimulate moral socialization at work by promoting social and moral skills and competencies (Weber et al., 2009). However, empirical research did not find evidence for this idea (Pircher Verdorfer and Weber, 2016). Our results indicate that perceived social impact may be an alternative mechanism linking SMC and prosocial employee behavior.

Consistent with Grant’s (2007) relational job design framework, empirical research has found that perceived social impact predicts prosocial work motivation (Castanheira et al., 2016; Ho and Wu, 2019) and prosocial behavior at work (Grant et al., 2007; Grant, 2008b). Building on the learning-generalization hypothesis (Kohn and Schooler, 1973) and its empirical evidence, we proposed that the perception of social impact may affect prosocial behavioral intentions beyond the work domain due to the generalization of perceived social impact. The findings provide initial support for this idea. By linking the work-related construct of perceived social impact with a prosocial intention outcome outside work, we complement the relational job design framework (2007) and its empirical support (Grant et al., 2007; Grant, 2008b; Castanheira et al., 2016; Ho and Wu, 2019).

Drawing on Karasek’s (2004) work on job socialization, we provided arguments for a spillover effect of SMC at work on prosocial behavior outside the work context and found initial evidence for such an effect. This study complements prior research indicating that the characteristics of work have long-term effects on personal attitudes (Weston et al., 2021), family life (Lee et al., 2019b), and leisure behavior (Staines, 1980). With respect to prosocial behavioral orientations, the results of our study support and extend previous findings on spillover effects of SMC (Weber et al., 2009; Pircher Verdorfer et al., 2013) by providing evidence for perceived social impact as an underlying mechanism. Considering that SMC is an indicator of the presence of participative organizational structures and practices (Weber et al., 2009; Pircher Verdorfer et al., 2013), the results of the study are consistent with recent meta-analytic findings on organizational democracy, suggesting that employees are socialized through participative organizational structures and practices to the extent that their prosocial behavioral orientations further develop (Weber et al., 2020).

### Practical Implications

This study provides organizations with guidance on how to increase the perception of social impact among their employees. Considering that employees who are aware of their social impact in the workplace are more likely to make efforts to help customers (Grant et al., 2007; Grant, 2008b) and to contribute to service quality and organizational success (Anderson and Zeithaml, 1984), the findings may be particularly relevant for organizations in the service sector. To develop high levels of SMC, organizations may implement interventions designed to increase cooperation, egalitarian communication, mutual care, and respect in the organization. In this respect, leadership interventions may be particularly effective because leaders play an important role in establishing and developing the organizational climate, including prosocial norms and values (Grojean et al., 2004).

The organizational democracy literature (Weber et al., 2020) and the just community approach as the foundation of the SMC concept suggest that it is important to not only rethink the role and behavior of leaders but also the hierarchical structures and principles of command and control in organizations. Concerns about the decline of participation in social organizations (Putnam, 2000) and the critique of privatism in modern Western societies (Power et al., 1989) point to the importance of a greater consideration of the just community approach in companies and democratic organization of work. Democratically organized companies that enable employee participation may lead to employees taking responsibility for others and becoming involved in societal issues.

Moreover, the findings of this study indicate that employees who feel that their work positively affects others’ lives have higher intentions to make responsible purchase decisions. Governments and nongovernmental organizations that aim to support responsible purchasing should include labor contexts in their campaigns, reminding employees that they are able to make a difference in the lives of others. Such an intervention is relatively easy to implement for employees in social professions (e.g., nurses and childcare workers) because their work offers many opportunities to affect others positively. In contrast, employees in other professions may have fewer opportunities to recognize their positive impact on the lives of others (e.g., warehouse workers). These employees may benefit most from interventions aimed at increasing perceived social impact.

### Limitations and Future Directions

As with other studies, this study is not without limitations. In this study, all variables were assessed using self-reports, which raises concerns about potential bias due to common method variance (Podsakoff et al., 2012). By using two waves of data collection, we followed Podsakoff et al.’s (2012) suggestion to mitigate common method bias by introducing a temporal separation between the predictor and outcome variables. Therefore, it seems relatively unlikely that the results are biased due to common method variance. Self-reports also carry the risk of social desirability bias, which raises the possibility that employees overreported their socially responsible purchase intentions. However, we assured the participants of their anonymity, which lowers concerns with social desirability. In addition, research suggests that forcing respondents to weigh tradeoffs between social and utilitarian product attributes may prevent overreporting of socially responsible purchasing behavior (Auger et al., 2008). To obtain more realistic reports of socially responsible purchasing intentions, we included this tradeoff in the introduction of the items (“I would spend more money on products if I had information that…”).

Additionally, it should be noted that the effects were relatively small in magnitude. However, small effect sizes are common in autoregressive models and should not be dismissed as trivial because controlling for temporal stability removes a large portion of the variance in the outcome variable (Adachi and Willoughby, 2015). Another explanation for the small effects is that we examined relationships between employees’ experiences at work and their behavioral intentions in the nonwork domain. Indeed, small effect sizes are common in spillover research (Maki et al., 2019). However, even small effects may have meaningful practical implications (Prentice and Miller, 1992).

On a related note, the relatively small effect sizes found in this study indicate that moderators may be present. The relational job design framework suggests that affective commitment to beneficiaries moderates the relationship between perceived social impact and the motivation to make a prosocial difference (Grant, 2007). That is, prosocial behavior is influenced not only by the perception of one’s impact but also by the extent to which one cares about the other person. Thus, the effect of perceived social impact on socially responsible purchase intention may vary depending on employees’ affective commitment to, for example, factory workers. Previous research has shown that people who feel more connected to others are more likely to believe that their actions have a substantial impact (Cojuharenco et al., 2016) and that feeling connected and emotionally bound to humanity predicts Fairtrade consumption (Reese and Kohlmann, 2015). Therefore, we recommend that further research on responsible purchasing takes a closer look at the temporal dynamics and reciprocal effects of employees’ feelings of connectedness and their perceptions of (social) impact.

We argued that the association between perceived social impact and socially responsible purchase intention is due to generalization processes from specific (i.e., work-related) perceived social impact to more global assessments of one’s positive impact on others. The finding of a significant relationship between perceived social impact and socially responsible purchase intention does not directly support the notion that generalization is the underlying process, as we did not assess employees’ general perceptions of social impact. In fact, the effect of perceived social impact on socially responsible purchase intention may occur through processes other than generalization. For example, Sonnentag and Grant (2012) conducted a daily diary study with rescue workers and found that the experience of helping others at work was positively related to positive affectivity at bedtime. Considering that responsible purchasing has been shown to be strongly influenced by consumers’ positive emotional states (Ladhari and Tchetgna, 2017), future studies may explore the role of positive affectivity in the relationship between perceived social impact and responsible purchasing.

In addition to the mediating effect *via* perceived social impact, we found a direct effect of SMC on socially responsible purchase intention, suggesting that other processes may explain the effect. Future research might examine moral values and emotions as mechanisms underlying the relationship between SMC and socially responsible purchasing. For example, previous studies have shown that moral elevation – the feeling of warmth and expansion in response to other people’s goodness, kindness, and compassion (Haidt, 2003) – is an important mechanism underlying associations between prosocial contexts and prosocial intentions (Pohling et al., 2019).

More research is needed that investigates the effects of SMC over time. The only other longitudinal study thus far has examined the effects of SMC on the applicability of signature character strengths at work 6 months later (Höge et al., 2020). Previous studies on the potential spillover effects of SMC on prosocial attitudes and behavioral intentions beyond the work context have used cross-sectional data (Weber et al., 2009; Pircher Verdorfer et al., 2013). Although this study provides initial longitudinal evidence for the spillover of SMC, future research should aim to provide more in-depth insights into the temporal dynamics of SMC and employees’ experiences and behavior outside the work context. Because there was no theoretical basis for defining optimal time lags between the measurements of the variables examined in this study, we followed the job socialization research (Bauer et al., 1998; Woodrow and Guest, 2020) and used two measurement points with a time lag of 12 months. Future studies should use multiple measurement points to examine when the effects of SMC on employee prosociality occur. This knowledge will be particularly useful for researchers investigating the effects of organizational interventions designed to increase SMC.

## Conclusion

This study sought to gain an in-depth understanding of the process by which SMC in organizations spills over to employees’ prosocial behavioral intentions outside work. We examined the relationships between SMC, perceived social impact, and socially responsible purchase intention over a time period of 1 year and tested the indirect relationship between SMC and socially responsible purchase intention *via* perceived social impact. The results showed that SMC was positively related to perceived social impact, which, in turn, was positively related to employees’ socially responsible purchase intention. The study results suggest that experiences at work may affect employees’ socially responsible purchasing in their private lives. This study advances research on SMC and the relational job design framework by providing evidence for longitudinal relationships of their core constructs in the context of responsible purchasing. The findings provide organizations with guidance on how to increase employees’ perceptions of social impact and their responsible purchasing.

## Data Availability Statement

The raw data supporting the conclusions of this article will be made available by the authors, without undue reservation.

## Ethics Statement

This study was carried out in accordance with the recommendations of the German Psychological Society (DGPs) guidelines and the Local Ethics Committee of the Faculty of Psychology and Human Movement at the University of Hamburg. The protocol was approved by the Local Ethics Committee of the Faculty of Psychology and Human Movement at the University of Hamburg. The participants provided their written informed consent to participate in this study.

## Author Contributions

MSc, GT, CB, and EB contributed to the conception and design of the study. MSc organized the database, performed the statistical analyses, and wrote the first draft of the manuscript. MSt contributed to the data analysis. MSt, GT, CB, and EB contributed to the manuscript revision, and read and approved the submitted version. All authors contributed to the article and approved the submitted version.

### Conflict of Interest

The authors declare that the research was conducted in the absence of any commercial or financial relationships that could be construed as a potential conflict of interest.

## Footnotes

^1^Fit indices of the model, including gender, were *χ*^2^ (1) = 9.35, *p* < 0.01; TLI = 0.81; CFI = 0.99; SRMR = 0.03; RMSEA = 0.13, 90% CI [0.06, 0.21]. Standardized path coefficients controlling for temporal stability effects of the respective variables: path *a* (*b* = 0.29, *SE* = 0.07, 95% CI [0.16, 0.42]), path *b* (*b* = 0.09, *SE* = 0.04, 95% CI [0.01, 0.17]), direct effect path *c’* (*b* = 0.22, *SE* = 0.07, 95% CI [0.09, 0.37]), indirect effect *ab* (*b* = 0.03, *SE* = 0.01, 95% CI [0.00, 0.05]), total effect *c* (*b* = 0.25, *SE* = 0.07, 95% CI [0.12, 0.39]).

## Appendix

**German Items of the Socio-Moral Climate Scale (Pircher Verdorfer et al., 2015):**

Wenn es bei uns unterschiedliche Ansichten bei wichtigen Angelegenheiten gibt, wird offen damit umgegangen.

Bei uns geht man offen mit Konflikten und Interessensgegensätzen um.

Wenn es zu Spannungen zwischen Unternehmensinteressen und den Interessen der Arbeitenden kommt, sprechen alle Beteiligten offen darüber.

Wenn hier jemand ungerecht behandelt oder übergangen wird, so wird dies offen angesprochen.

Man kann bei uns auch Fehler machen, ohne dafür bestraft zu werden.

Gegenseitiger Respekt wird bei uns groß geschrieben.

Das Vertrauen untereinander lässt bei uns einiges zu wünschen übrig.

Die Mitarbeiter*innen werden unabhängig von der Ausbildung und Qualifikation geachtet.

Niemand muss sich hier ein Blatt vor den Mund nehmen; jeder kann sich offen zu Meinungen von Entscheidungsträgern äußern, ohne negative Folgen fürchten zu müssen.

Mitarbeiter*innen werden ermutigt und bestärkt, den eigenen moralischen Standpunkt hinsichtlich Vorgehen und Vorhaben des Unternehmens zu äußern.

Auch bei weit reichenden Veränderungen im Unternehmen haben die Mitarbeiter*innen ein Wörtchen mitzureden.

Wichtige Entscheidungen, die bei uns getroffen werden, beruhen auf der Meinung einiger Weniger.

Bei uns gibt es kaum „heilige Kühe“. Es ist möglich, Prinzipien in Frage zu stellen, falls sie für den gemeinsamen Erfolg oder die gute Zusammenarbeit nicht mehr geeignet sind.

Bei uns wird jeder - seinen Fähigkeiten entsprechend - auch mit schwierigen Aufgaben betraut.

Das Vertrauen der Führungspersonen in die Fähigkeit der Mitarbeiter*innen, verantwortlich zu handeln, ist nicht sehr groß.

Es wird bei uns gefördert, dass man sich für andere einsetzt.

Wer sich dazu berufen fühlt, dem wird bei uns Verantwortung für andere Kolleginnen/Kollegen übertragen.

Den Bedürfnissen aller Mitarbeiter*innen gerecht zu werden ist eine echte Herausforderung, aber es wird bei uns ernsthaft versucht.

Wenn jemand bei uns persönliche Probleme hat, kann er mit dem Verständnis der Anderen rechnen.

Auf persönliche Gefühle der Mitarbeiter*innen wird bei uns wenig Rücksicht genommen.

Bei wichtigen Entscheidungen berücksichtigen die Verantwortlichen normalerweise das Wohl der betroffenen Mitarbeiter*innen.

**German Translation of the Perceived Social Impact Scale (Grant, 2008b):**

Ich bin mir der positiven Auswirkungen, die meine Arbeit auf andere Menschen hat, sehr bewusst.

Ich bin mir sehr darüber bewusst, auf welche Weise meine Arbeit anderen Menschen zugutekommt.

Ich habe das Gefühl, dass ich durch meine Arbeit einen positiven Einfluss auf andere haben kann.

**Socially Responsible Purchase Intention Scale (Adapted and Translated From Balderjahn et al., 2013):**

*Ich würde mehr Geld für Produkte ausgeben wenn mir Informationen darüber vorliegen würden, dass bei der Herstellung…*

die Rechte der Arbeitnehmer*innen eingehalten wurden.

kein Arbeitnehmer*innen zur Zwangsarbeit verpflichtet wurde.

keine Kinder beteiligt gewesen sind.

Arbeitnehmer*innen nicht diskriminiert wurden.

die Arbeitsbedingungen den internationalen gesetzlichen Standards entsprachen.

die Arbeitnehmer*innen gerecht bzw. Fair entlohnt wurden.
